# Validation of a genetic risk score for atrial fibrillation: A prospective multicenter cohort study

**DOI:** 10.1371/journal.pmed.1002525

**Published:** 2018-03-13

**Authors:** Evan D. Muse, Nathan E. Wineinger, Emily G. Spencer, Melissa Peters, Riley Henderson, Yunyue Zhang, Paddy M. Barrett, Steven P. Rivera, Jay G. Wohlgemuth, James J. Devlin, Dov Shiffman, Eric J. Topol

**Affiliations:** 1 Scripps Translational Science Institute, The Scripps Research Institute, La Jolla, California, United States of America; 2 Division of Cardiovascular Disease, Scripps Clinic–Scripps Health, La Jolla, California, United States of America; 3 Quest Diagnostics, San Juan Capistrano, California, United States of America; University of Oxford, UNITED KINGDOM

## Abstract

**Background:**

Atrial fibrillation (AF) is the most commonly encountered arrhythmia and is associated with an elevated risk of stroke. Improving the identification of patients with the highest risk for AF to enable appropriate surveillance and treatment, if necessary, is critical to reducing AF-associated morbidity and mortality. Multiple common single nucleotide polymorphisms (SNPs) are unequivocally associated with the lifetime risk of AF. In the current study we aimed to prospectively validate an AF genetic risk score (GRS) in previously undiagnosed patients at risk for AF.

**Methods and findings:**

Individuals 40 years of age or older with 1 clinical risk factor for AF, presenting with symptoms of AF, or with a first diagnosis of AF, were enrolled for genetic testing and ambulatory cardiac rhythm monitoring with an adhesive patch monitor or a long-term Holter monitor (mean wear time 10 days 21 hours and 13 days 18 hours, respectively). An AF event was the first diagnosis of AF by ECG, patch monitor, or long-term Holter monitor. The AF GRS was determined for each participant based on the weighted contribution of 12 genetic risk loci. Of 904 participants, 85 manifested AF. Their mean age was 66.2 (SD 11.8) years; 38% of participants were male. Participants in the highest quintile of AF GRS were more likely (odds ratio 3.11; 95% CI 1.27–7.58; *p =* 0.01) to have had an AF event than participants in the lowest quintile after adjusting for age, sex, smoking status, BMI, hypertension, diabetes mellitus, heart failure, and prior myocardial infarction. Study limitations included an ethnically homogenous population, a restricted rhythm monitoring period, and the evolving discovery of SNPs associated with AF.

**Conclusions:**

Prospective assessment of a GRS for AF identified participants with elevated risk of AF beyond established clinical criteria. Accordingly, a GRS for AF could be incorporated into overall risk assessment to better identify patients at the highest risk of developing AF, although further testing in larger populations is needed to confirm these findings.

**Trial registration:**

ClinicalTrials.gov NCT01970969

## Abstract 2

In prospective cohort study, Eric Topol and colleague validate a genetic risk score for identification of patients at the highest risk of developing atrial fibrillation.

## Author summary

**Why was this study done?:**

- Atrial fibrillation (AF) is a common heart rhythm disturbance that can lead to devastating strokes.
- While clinical factors such as age, high blood pressure, and obesity can increase the chances of developing AF, several genetic determinants of AF also play a role.
- Here we assessed the ability of a genetic risk score (GRS) for AF to identify individuals presenting with symptoms with the highest likelihood of AF on cardiac rhythm monitoring.

**What did the researchers do and find?:**

- Individuals 40 years of age or older with at least 1 clinical risk factor for AF presenting either with symptoms of AF or with the first diagnosis of AF on electrocardiogram were enrolled.
- A patch-based or long-term Holter cardiac rhythm monitor was fitted to individuals without AF on electrocardiogram, and they were monitored for upwards of 2 weeks.
- DNA was isolated from a blood sample, and an AF GRS was calculated for each participant.
- We found that individuals with the highest AF GRSs were 3 times more likely to be diagnosed with AF during the study than participants with the lowest AF GRSs.

**What do these findings mean?:**

- An AF GRS may be incorporated into an overall risk assessment strategy to better identify patients at the highest risk of developing AF.
- For patients with stroke of unknown origin, a high AF GRS may be helpful to guide diagnostic testing.
- In the future, individuals with high AF genomic risk may be able to use this information to help prevent the arrhythmia from occurring.

## Introduction

Stroke remains the fifth leading cause of death in the United States, despite increased recognition and better management of overall cardiovascular disease risk, which has led to a decline in stroke mortality [1]. More than 750,000 people experience a stroke event each year in the US, with a prevalence of 2.6% in the US adult population [1]. While conventional risk factors for stroke—including cigarette smoking, hypertension, diabetes mellitus, dyslipidemia, and heart failure—account for a sizable portion of stroke episodes, atrial fibrillation (AF) is associated with a quarter of all stroke events [2–4].

AF is the most common pathological cardiac arrhythmia, present in nearly 6 million people in the US alone, with the prevalence estimated to grow to over 12 million people by 2030 [5]. It is not uncommon for AF to remain undetected in asymptomatic individuals. AF has been documented in asymptomatic individuals not only in patients with implanted cardiac devices [6,7], but also in population-based screening studies using smartphone electrocardiogram (ECG) sensors [8–11]. These efforts to better diagnose subclinical, asymptomatic AF may be useful for implementing appropriate therapies to reduce the risk of stroke [12].

To this end, efforts have been made to predict the occurrence of AF using risk scores based on clinical factors associated with disease [13,14]. While clinical risk factors for AF (e.g., obesity, hypertension, and obstructive sleep apnea) are well established, risk scores incorporating these factors have fallen short in validation studies [15]. Therefore, efforts continue to improve risk prediction by incorporating genetic risk factors. Genetic factors are estimated to account for ~40% of a person’s total AF risk [16], and genome-wide association studies (GWASs) of increasing size have identified multiple single nucleotide polymorphisms (SNPs) associated with AF on the genome-wide level [17–23]. For AF, several genetic risk scores (GRSs) have been characterized and tested in various studies, illustrating their ability to identify individuals at increased risk of AF [24–29]. But some of these findings pertain to a lifetime risk of AF or >14 years of follow-up, and have not yet provided any insight on immediate risk for an individual with established risk factors presenting with symptoms.

Here, in this multicenter study of symptomatic participants without prior diagnosis of AF, we aimed to prospectively validate a 12-SNP AF GRS for identifying patients at an increased risk for AF during 2 weeks of ambulatory cardiac rhythm monitoring.

## Methods

### Participant recruitment and inclusion and exclusion criteria

The study was approved by the institutional review board (IRB) at the Scripps Clinic (La Jolla, CA), which provided overall study oversight, in addition to the individual IRBs affiliated with each recruitment center. Participants were enrolled from December 2, 2013, through January 19, 2016. The complete list of recruitment centers is provided in S1 Table. Informed consent was provided by each patient prior to enrollment. Patients presenting with any symptoms suggestive of AF determined by the clinician to necessitate ambulatory cardiac rhythm monitoring were evaluated for inclusion in this study. Patients were eligible for enrollment if they were 40 years of age or older and were capable of providing informed consent in addition to providing a blood sample for genetic analysis. Study participants were also required to have at least 1 of the following clinical characteristics: hypertension, ischemic stroke with no defined etiology within the past 6 months, high BMI (>30 kg/m^2^), history of heart failure, clinically significant murmur, first degree atrioventricular block (PR interval > 200 ms), chronic kidney disease, hypertrophic cardiomyopathy, congenital heart disease, chronic obstructive pulmonary disease, obstructive sleep apnea, diabetes mellitus, regular excess alcohol consumption (males >14 drinks/week, females >7 drinks/week), or a family history of AF. Patients with a prior diagnosis of AF or atrial flutter were excluded. Additionally, patients with cardiac surgery (coronary artery bypass grafting, valve replacement or repair, pericardial stripping, etc.) within the previous 30 days, with hyperthyroidism, receiving permanent pacing therapy, with skin allergies or sensitivities to adhesives, or who were anticipated to have exposure to high frequency surgical equipment during the monitoring period were not eligible for enrollment. An independent data and safety monitoring board provided ongoing study oversight throughout the trial period. This study (S1 Protocol) was registered with ClinicalTrials.gov (NCT01970969) and is reported as per the TRIPOD guidelines (S1 Checklist).

### Cardiac rhythm monitoring and event adjudication

The primary event was an instance of AF/atrial flutter as defined by standard electrocardiographic criteria for a minimum duration of 5 seconds (AF event). AF events were assessed either by ambulatory cardiac rhythm monitoring or on 12-lead ECG. Patients meeting the specified inclusion criteria presenting to an outpatient clinic for evaluation of symptoms with high clinical suspicion for AF were either provided an adhesive patch monitor (Zio patch, iRhythm Technologies), if enrolled at centers within the US, or fitted with a long-term Holter monitor (Spiderflash, Soren Group) at Canadian centers. Participants were instructed to wear the cardiac rhythm monitor for the life of the device (~2 weeks). Symptomatic patients presenting with the first diagnosis of AF on 12-lead ECG were not required to have additional ambulatory cardiac rhythm monitoring. Events that were considered possible occurrences of AF were independently adjudicated by 2 separate physicians with expertise in cardiac rhythm interpretation, with a third physician’s review available for incongruent cases.

### Genotyping

DNA was extracted from whole blood using the MagNA Pure 96 instrument and reagent kit (Roche Life Science). SNaPshot multiplex genotyping (Thermo Fisher Scientific) was used to simultaneously genotype 12 SNPs comprising the AF GRS (Table 1).

**Table 1:** Single nucleotide polymorphisms associated with AF used for the determination of the AF GRS.

Locus	Gene	SNP	Modeled allele	MAF	Weight
1q21	*KCNN3*	rs13376333	T	0.30	0.12
1q24	*PRRX1*	rs3903239	G	0.40	0.13
4q25	*PITX2*	rs10033464	T	0.10	0.33
4q25	*PITX2*	rs17570669	T	0.07	−0.31
4q25	*PITX2*	rs2200733	T	0.13	0.54
4q25	*PITX2*	rs3853445	C	0.27	−0.15
7q31	*CAV1*	rs3807989	A	0.42	−0.11
9q22	*C9orf3*	rs10821415	A	0.40	0.10
10q22	*SYNPO2L*	rs10824026	G	0.18	−0.14
14q23	*SYNE2*	rs1152591	A	0.46	0.12
15q24	*HCN4*	rs7164883	G	0.19	0.17
16q22	*ZFHX3*	rs2106261	T	0.19	0.22

AF, atrial fibrillation; GRS, genetic risk score; MAF, mean allele frequency; SNP, single nucleotide polymorphism.

### Modeling of GRS and statistical analyses

Comparisons of baseline characteristics between patients with and without detected AF were conducted using chi-squared and *t* tests for categorical and quantitative traits, respectively. We sought to validate an existing 12-SNP AF GRS that had previously been examined in a retrospective manner [27]. The AF GRS in this study was calculated using a weighted allele-counting approach from previously reported weights [27] (Table 1). Expected AF events in the 2 groups were calculated using reference to previous studies [30,31]. We expected roughly 80 AF events in 1,000 at-risk patients. Using this event rate, and based on a simulation in which the absolute rate of AF increased by 2% per AF GRS quintile (AF rate is half the population average in the lowest quintile and 1.5 times the population average in the highest quintile), we had 90% power to detect an association between AF GRS quintile and AF at an alpha of 5% (S1 Text). To account for the small number of missing genotype data (15 genotypes, 0.14% missing rate across all markers), missing genotypes were replaced with the average genotype at that marker based on additive coding. The association between AF GRS and newly detected AF was assessed using logistic regression. Two approaches to model AF GRS were used: (1) coding the AF GRS as a quantitative, continuous variable and (2) coding the AF GRS as a quantitative variable using quintile categories. Results are presented conditional (adjusted) and not conditional (unadjusted) for the following covariates: age, sex, smoking status, BMI, the presence of diabetes mellitus, and history of hypertension, myocardial infarction, and heart failure. All analyses were performed in R version 3.2.3 (R Project for Statistical Computing).

## Results

### Patient characteristics

A total of 934 participants were enrolled from December 2, 2013, through January 19, 2016. The final analysis excluded 30 participants who did not provide a blood sample for genotyping, were missing adhesive patch monitor data, or had a prior diagnosis of AF identified upon chart review. Accounting for these exclusions, the final analysis included 904 participants. A total of 85 participants were discovered to have AF from the adhesive patch monitor (*n* = 44) or ECG (*n* = 41) during the study. The mean wear time was 10 days and 21 hours for the adhesive patch monitors and 13 days and 18 hours for the long-term Holter monitors. There was no difference in wear time between participants with and without the diagnosis of AF (*p =* 0.49). The primary presenting indication for which cardiac rhythm monitoring was pursued was palpitations/tachycardia (72%), followed by syncope/near-syncope (14%), transient ischemic attack/stroke (9%), and chest pain/dyspnea (5%).

Baseline characteristics for participants with and without AF are provided in Table 2. The self-reported ethnicity of participants was predominantly white (92.6%), with other ethnicities including African-American (5.2%), Asian (1.7%), and Native American (0.2%). The mean age for participants with AF (68.5 years [SD 11.2]) was greater than for participants without AF (65.9 years [SD 11.8], *p =* 0.046). Men made up the majority of participants with AF (52%) and the minority for participants without AF (36%). Participants with AF had a higher mean BMI (31.7 kg/m^2^) than the group without AF (29.4 kg/m^2^, *p =* 0.02), but the prevalence of diabetes was lower (13% for those with AF compared with 23% for those without AF, *p =* 0.04). There were no intergroup differences in the prevalence of the other comorbidities evaluated. Additionally, the stroke risk as evaluated by CHADS_2_ and CHA_2_DS_2_-VASc scores did not differ between the 2 groups.

**Table 2:** Baseline characteristics.

Characteristic	No AF (*n* = 819)	AF (*n* = 85)	*p*-Value
Age	65.9 (11.8)	68.5 (11.2)	0.05
Male	297 (36%)	44 (52%)	0.007
White	755 (92%)	82 (96%)	0.22
Current smoker	78 (9.5%)	6 (7.1%)	0.58
Height (m)	1.69 (0.10)	1.72 (0.12)	0.04
Weight (kg)	83.9 (20.9)	93.4 (26.6)	0.002
BMI (kg/m^2^)	29.4 (6.6)	31.7 (8.5)	0.02
Hypertension	626 (76%)	67 (79%)	0.72
BMI > 30 kg/m^2^	305 (37%)	44 (52%)	0.01
Hypothyroidism	194 (24%)	15 (18%)	0.26
Diabetes mellitus	190 (23%)	11 (13%)	0.04
Obstructive sleep apnea	157 (19%)	22 (26%)	0.18
Family history of AF	137 (17%)	20 (24%)	0.15
History of MI	73 (9%)	7 (8%)	0.99
Ischemic stroke or TIA (within past 6 months)	72 (9%)	6 (7%)	0.73
Chronic obstructive pulmonary disease	77 (8%)	10 (12%)	0.61
Excess alcohol consumption	63 (8%)	12 (14%)	0.07
History of heart failure	41 (5%)	8 (9%)	0.15
Chronic kidney disease	36 (4%)	5 (6%)	0.72
CHADS2 score	1.6 (1.2)	1.6 (1.2)	0.72
CHADS2VASc score	2.9 (1.6)	2.8 (1.6)	0.46
Patch monitor wear time, mean	10 d 21 h	11 d 4 h	0.49

Data are given as mean (SD) or *n* (percent), unless otherwise indicated.

AF, atrial fibrillation; BMI, body mass index; MI, myocardial infarction; SD, standard deviation; TIA, transient ischemic attack.

### AF GRS and AF events

The AF GRS was calculated as a weighted sum of the 12 SNPs (Table 1) for each participant based on their genotype for these SNPS, and the scores ranged from −0.84 to 1.78. The mean AF GRS for the 85 participants diagnosed with AF during the study was significantly higher than the mean AF GRS for the 819 participants without AF (mean [SD]: 0.582 [0.378] versus 0.439 [0.382], *p =* 0.001). The incidence of AF increased across increasing quintiles of AF GRS (Table 3), with the odds per SD increase in AF GRS increasing by a factor of 1.43 (95% CI 1.15–1.77; *p =* 0.001). In an unadjusted analysis, participants in the highest quintile of AF GRS (>0.77) had an increased risk of having an AF event during the course of the study (odds ratio [OR] 2.83; 95% CI 1.21–6.61; *p =* 0.02) as compared with participants in the lowest AF GRS quintile. After adjusting for age, sex, smoking status, BMI, diabetes mellitus, hypertension, prior myocardial infarction, and heart failure, the odds per SD increase in AF GRS increased by a factor of 1.45 (95% CI 1.16–1.83; *p =* 0.001), with participants in the highest AF GRS quintile having greater than 3 times the odds (OR 3.11; 95% CI 1.27–7.58; *p =* 0.01) of AF diagnosis compared with those at lowest AF GRS quintile (Table 3). Modeling AF GRS increased the C-statistic from 0.687 to 0.719 over a model that considered only age, sex, smoking status, BMI, diabetes mellitus, hypertension, prior myocardial infarction, and heart failure (*p =* 0.001). Additionally, when the AF GRS was assessed according to GRS tertiles, the highest AF GRS tertile had greater than 2 times the risk of an AF event compared to the lowest tertile (unadjusted OR 2.26; 95% CI 1.27–4.01; *p <* 0.01; adjusted OR 2.32; 95% CI 1.28–4.20; *p =* 0.006). Alternative time-to-event approaches are not presented due to the short AF monitoring period, though the results were consistent with the logisticregression results.

**Table 3:** Risk estimates of AF events according to AF GRS quintile (12 SNPs).

AF GRS quintile	Unadjusted OR (95% CI)	*p*-Value	Adjusted* OR (95% CI)	*p*-Value
1 (*n* = 187)	Reference	—	Reference	—
2 (*n* = 177)	2.35 (0.94–5.87)	0.07	2.37 (0.89–6.30)	0.08
3 (*n* = 180)	2.48 (1.05–5.87)	0.04	2.47 (0.98–6.22)	0.054
4 (*n* = 182)	3.40 (1.48–7.78)	0.004	3.49 (1.48–8.23)	0.005
5 (*n* = 178)	2.83 (1.21–6.61)	0.02	3.11 (1.27–7.58)	0.013

AF GRS quintile ranges: Q1 ≤ 0.14; Q2 > 0.14 and ≤ 0.33; Q3 > 0.33 and ≤ 0.50; Q4 > 0.50 and ≤ 0.77; Q5 > 0.77.

*Adjusted by age, sex, smoking status, BMI, diabetes, hypertension, prior myocardial infarction, and heart failure.

AF, atrial fibrillation; GRS, genetic risk score; CI, confidence interval; OR, odds ratio.

In an effort to examine the individual contributions of the 12 SNPs that composed the AF GRS, we evaluated the association of each SNP with AF in both unadjusted and adjusted models (S2 Table), though this study was not well powered to detect individual marker effects. Eleven of the 12 SNPs were consistent with prior results: either the direction of the effect allele was consistent with previous reports, or the result was not nominally significant (*p <* 0.05). However, rs10033464 (4q25, *PITX2*) displayed a risk estimate in the opposite direction than had been previously reported by others. This discrepancy appears to be in part due to linkage disequilibrium between this marker and the nearby SNP with the strongest association in this gene, rs2200733 (*p =* 0.005). We and others have observed that the risk alleles at these markers are negatively associated with each other. There remains uncertainty in the optimal genetic risk model for AF, and superior models will certainly evolve in the future as more genetic discoveries are made. In this light, we also considered a modified AF GRS that included only the most strongly associated SNP in each gene—which is a common solution in GRS estimation to eliminate confounding due to linkage disequilibrium [32]. The resulting 9-SNP model included rs2200733 for *PITX2* and the other 8 SNPs from the other genes. The risk of AF with this modified AF GRS, as in the 12-SNP model, was greater for the highest AF GRS quintile than for the lowest AF GRS quintile (OR 5.07; 95% CI 2.04–12.60; *p* < 0.001), and this model appeared to better differentiate risk of AF throughout the distribution of genetic risk (S3 Table). As expected, the risk estimates derived for both AF GRS models remained significant in an analysis limited to participants who self-reported as white (S4 Table).

## Discussion

We aimed to assess the ability of a previously developed AF GRS comprising 12 common SNPs (minor allele frequency > 5%) that have been found to be associated with AF at a genome-wide significance level to identify patients at the highest risk of developing AF in a prospective manner. Indeed, in this cohort of 904 participants of predominantly white ethnicity, 85 had AF events, and participants in the highest quintile of AF GRS had a risk greater than 3 times that of participants in the lowest AF GRS quintile. These findings are consistent with the original report [27] of this specific AF GRS, in addition to other studies utilizing a multi-allele GRS [26,28]; however, all of these studies were conducted on a retrospective basis.

By harnessing advanced technology to realize the benefits of long-term cardiac rhythm monitoring using an adhesive patch monitor in lieu of the dated 24-hour Holter monitor [33], participants were monitored for over 10 days after presenting to a clinic with specific symptoms prompting cardiac rhythm monitoring with a suspicion for AF. It has become clear that extended periods of ambulatory cardiac rhythm monitoring increase the yield of AF detection, especially in patients with cryptogenic stroke [34]. AF is the most common arrhythmia, and with age being an important risk factor, its prevalence is estimated to increase steadily as humans live longer and with more chronic disease. While documented AF accounts for 1 out of every 6 strokes, a quarter of all strokes are initially categorized as “cryptogenic” with no identifiable cause. With prolonged rhythm monitoring strategies, many of these cryptogenic strokes are subsequently attributed to AF [12,34–36]. An AF GRS may help identify individuals at the highest risk of subclinical and paroxysmal AF who would benefit from a cardiac rhythm monitoring strategy even before a devastating stoke event. Improving identification of patients with AF, and initiating appropriate anticoagulant therapies when appropriate, is important given the risk of embolic stroke, which is more likely to be associated with major neurological deficits than non-embolic stroke [37].

To date, the relative risk of AF associated with individual SNPs ranges from just above 1 to a highest value of 1.64 (rs6817105, *PITX2*). Thus, the ability to combine common SNPs into an AF GRS with the ability to differentiate a greater than 3-fold increased risk of AF in a population on a prospective basis may be useful. Such a panel of SNPs could be assayed at low cost and be used in conjunction with an evaluation of a patient with possible AF. For example, even 10 days of patch-based monitoring may not yield the diagnosis of subclinical AF and indicate that additional monitoring would be useful [34,35].

Several limitations of our study should be examined. Despite our efforts to recruit patients from various geographic regions across the US and Canada ranging from rural to urban settings, the vast majority of participants (93%) self-reported as white, with only minor representation from other ethnicities. The bulk of our understanding of genetic variants in association with disease has come from GWASs in populations of European ancestry, such that considerable assessment in other ancestries is important, and the 12-SNP panel we used in the study cannot be extrapolated beyond those of European ancestry [38,39]. With the inclusion of more ethnically diverse populations in genetic studies, the importance of ethnicity in determining the association of SNPs with disease will be magnified and will certainly need to be accounted for in future GRS calculations [40,41]. It was recently shown that rs10824026 (10q22), 1 of the SNPs utilized in the current AF GRS, conferred a greater risk for AF in white individuals than in black individuals [42]. Although the current study is underpowered to assess AF risk by ethnicity, self-identified African-American participants in this study were far more likely to carry the risk allele (G) (53% GA and 38% GG) compared to self-identified white participants (26% GA and 2% GG).

Since the initiation of our study, additional SNPs associated with AF have been identified, and we expect this trend to continue as the genetic datasets expand. Our AF GRS did not include 5 recently discovered SNPs [43], though 1 of these SNPs was specific for a Japanese population and the others had lower relative risks than the SNPs already included in our AF GRS. Additionally, our preliminary finding that demonstrated greater differentiation in AF based on a 9-SNP (1 SNP per gene) model simply shows that improvements can be made. Future efforts in developing polygenetic risk predictors of AF should focus on optimizing the set of markers and, potentially, population-specific weights assigned to individual markers. Yet, improving the predictive performance of a risk score solely based on genetic data will reach an upper limit [44]. As additional AF biomarkers are determined through multi-omics approaches (proteomics, lipidomics, and metabolomics) or multiparameter physiological sensors, the addition of more complex clinical, physiological, and biomarker components to the AF GRS will be an exciting next step to developing a comprehensive AF risk score.

In terms of AF events, the mean monitoring time for patients evaluated with adhesive patch monitoring was less than 11 days, and there could have been patients for whom we did not capture their AF event during this time. Prolonged monitoring periods, as have been achieved using implantable monitoring devices, have been shown to detect increasingly more AF events over time, especially in patients with cryptogenic stroke [34–36]. Currently, there is little guidance regarding optimal length of monitoring. While we do not feel that the future of diagnostic ambulatory cardiac rhythm monitoring is grounded in expensive, implantable devices, this role may instead be filled using unobtrusive and passive wearables. A SNP-based AF GRS, however, provides a fixed overall lifetime genetic risk assessment for AF, and given the short-term follow-up time in the current study, we expect that the ability of the AF GRS to identify individuals with the highest AF risk would likely increase with a longer monitoring period and follow-up. Likewise, we illustrate the strength of a GRS-based approach to disease screening and demonstrate the ability of the AF GRS to temporally stratify symptomatic patients with the probability of an AF diagnosis during 2 weeks of rhythm monitoring. In doing so, we have moved from the typical retrospective GWAS, without temporal association, to a prospective GRS screen that leverages the genomic risk for an individual under evaluation.

In conclusion, we prospectively validated a GRS for AF that may prove useful in the diagnostic evaluation of certain individuals who are being assessed for subclinical presence of this arrhythmia, and potentially as a means to help its prevention in the future.

## Supporting information

S1 Checklist TRIPOD checklist. (PDF)

S1 Text Reported SNPs for study population. (DOCX)

S1 Protocol Study protocol. (PDF)

S1 Table US and Canadian patient recruitment centers. (PDF)

S2 Table Performance of the individual SNPs of the AF GRS. (PDF)

S3 Table Risk estimates of AF events according to AF GRS quintile (9-SNP model). (PDF)

S4 Table Risk estimates for both the 12-SNP and 9-SNP AF GRS. (PDF)

## Abbreviations

AF: atrial fibrillation
ECG: electrocardiogram
GRS: genetic risk score
GWAS: genome-wide association study
OR: odds ratio
SNP: single nucleotide polymorphism
