# Supplementary material for: Validation of a genetic risk score for atrial fibrillation: A prospective multicenter cohort study
Source: PLoS Med. 2018 Mar 13;15(3):e1002525. doi: 10.1371/journal.pmed.1002525 (PMC5849279; doi:10.1371/journal.pmed.1002525)
Supplement: S1 Text — (DOCX) [file pmed.1002525.s002.docx]

**Sample Size**

The sample size of our studied population differed from our original considerations. As such, to ensure appropriate power, we performed 1,000 simulations with a total sample size of 904 using a simple model, but one that is consistent with the known genetic basis and prevalence of AF. We simulated samples falling into one of five quintiles in which the rate of AF was 4%, 6%, 8%, 10%, and 12%, respectively. As each quintile was simulated to be equally likely, the average rate of AF was 8% (close to our observed estimate) with the top and bottom quintiles demonstrating a 50% increase or decrease in this rate, respectively. We then used a logistic regression to test if the quintile (numeric) was associated with AF status. The power was roughly 90% (varying up or down based on the simulation) at a 0.05 significance level. Similarly, looking at simply the bottom vs. top quintile and using a logistic regression, the power was roughly 80%. The simulation was performed in R. This is a post hoc power calculation. Originally the study was designed to differentiate genetic risk on fewer markers with larger effects with a goal of 1,000 samples and an 8% rate of AF. While we did not reach this sample size (would reduce power), we did observe more AF events (9.4%) and new markers were discovered since then (both would increase power). Overall, we feel that we were sufficiently powered, and that the result we observe is unlikely to be a chance observation as it largely functions as confirmation of prior genetic studies.
