# Supplementary material for: Validation of a genetic risk score for atrial fibrillation: A prospective multicenter cohort study
Source: PLoS Med. 2018 Mar 13;15(3):e1002525. doi: 10.1371/journal.pmed.1002525 (PMC5849279; doi:10.1371/journal.pmed.1002525)
Supplement: S1 Protocol — (PDF) [file pmed.1002525.s003.pdf]

The GIRAFFE Study:

Genomi**c** **R**isk Markers for **A**trial **F**ibrillation

Following **E**xtended Cardiac Rhythm Monitoring

Study Protocol

April 17, 2014

Sponsor:

Scripps Translational Science Institute

3344 N. Torrey Pines Court, Suite 300

La Jolla, CA 92037

Study Principal Investigator:

Eric Topol MD
Scripps Translational Science Institute
3344 N. Torrey Pines Court, Suite 300
La Jolla, CA 92037
858-554-5708
Fax: 858-546-9272

Study Co-Investigators:

Evan D. Muse, MD, PhD
Paddy Barrett, MD
Nicholas Schork, PhD
John Sninsky, PhD
Jay Wohlgemuth, MD
Ondrej Libger, PhD
James J. Devlin PhD

CONTENTS

1. PURPOSE 3

2. BACKGROUND 3

3. HYPOTHESIS 5

4. STUDY DESIGN 5

5. SAMPLE STORAGE AND ANALYSIS 8

6. STATISTICAL CONSIDERATIONS 9

7. INCLUSION AND EXCLUSION CRITERIA 9

8. POSSIBLE BENEFITS 10

9. POSSIBLE RISKS AND ANALYSIS OF RISK/BENEFIT RATIO 11

10. RISK MANAGEMENT PROCEDURES 11

11. RECRUITMENT 13

12. SUBJECT PAYMENT/COSTS 13

13. ESTIMATED DURATION OF STUDY 13

14. CONSENT PROCEDURES 14

15. PRIVACY 14

16. DATA SECURITY 14

17. SUBJECT WITHDRAWAL 14

18. MONITORING 15

19. RECORD RETENTION 16

20. PUBLICATION 16

21. FACILITIES AND PERSONNEL 16

BIBLIOGRAPHY 16

APPENDIX 1 ZIO PATCH PATENT DIARY AND INSTRUCTIONS 21
APPENDIX 2 SAMPLE ZIO® PATCH NOTIFICATION CRITERIA 25
APPENDIX 3 SAMPLE ZIO® PATCH REPORT 26
APPENDIX 4 CLINICAL INFORMATION ON CASE REPORT FORM 27
APPENDIX 5 IRHYTHM ZIO® PATCH VARIABLES 29
APPENDIX 6 PATIENT RECRUITMENT FLYER 30
APPENDIX 7 SORIN SPIDERFLASH-T™ LOOP RECORDER INFORMATION 31
APPENDIX 8 SORIN SPIDERFLASH-T™ LOOP RECORDER USAGE ADVICE AND PATIENT DIARY 36

1. Purpose

This study will investigate the association between a set of single nucleotide polymorphisms (SNPs) and atrial fibrillation in patients at high risk of developing atrial fibrillation. The SNPs investigated will have been previously shown to be associated with the atrial fibrillation.

2. Background

Atrial fibrillation is the most commonly encountered arrhythmia [1, 2] and when documented is predicted to be responsible for approximately 25% of strokes [3-5]. However, 25% of persons who suffer an ischemic stroke have no cause identified [3, 4, 6, 7]. In reality, many of these strokes are likely to be accounted for by atrial fibrillation [7, 8]. With 30 - 40% of ischemic strokes being classified as cryptogenic [9-14] it is imperative that greater efforts be taken to diagnose and treat those strokes that are due to treatable conditions such as atrial fibrillation. Because subclinical atrial fibrillation goes unrecognized by the patient it could be said to pose the greatest risk. Figures vary as to the prevalence of subclinical atrial fibrillation but implanted cardiac device studies in those without previously documented atrial fibrillation suggest it is indeed common [15, 16] and carries an increased risk of stroke or systemic embolism [3]. With recent studies highlighting the potential deficiencies of aspirin treatment in atrial fibrillation [17] it has become critical that we accurately identify all subgroups of atrial fibrillation and initiate appropriate treatment to reduce the risk of stroke.

Although atrial fibrillation can follow a familial pattern [18] more defined screening strategies need to be identified to target non-familial atrial fibrillation. To date, several genome-wide association studies (GWAS) have identified and replicated a panel of SNPs that occur more commonly in persons with atrial fibrillation. The SNPs with the most predictive utility are rs2200733 and rs10033464 on chromosome 4q25 [1, 19-27], rs2106261 on chromosome 16q22 [20, 21], and rs13376333 on chromosome 1q21 [22]. Several other SNPs have also had been associated with atrial fibrillation at a GWAS level ($P<5X10^{-8}$) [47].

Of those SNPs on chromosome 4q25, rs2200733 was first reported to have an odds ratio of 1.84 for atrial fibrillation (1.51 - 2.23, $P = 9.8 X 10^{-10}$) and rs10033464 an odds ratio of 1.30 (1.00 -

1.69, P = 0.052) in the United States population [1], and when combined with other populations of European ancestry the association of rs10033464 moved well beyond the threshold of genome wide significance (OR = 1.39, P = 6.9 X 10^{-11}) [1]. With multiplicative models, the combined population attributable-risk (PAR) of both variants is approximate 20% in European ancestral populations [1]. It is estimated that approximately 35% of persons of European heritage have at least one of the variants [1]. Increasing copy number also confers a greater risk of atrial fibrillation [1]. Interestingly, the age at diagnosis of atrial fibrillation occurs between 1.10 and 2.28 (P = 1.29 X 10^{-6}) years earlier in those who carry the risk variants. Although both variants reside in a linkage disequilibrium (LD) block that do not contain any genes, the closest gene, PITX2, is located in an upstream LD block and is important in myocardial development because it directs asymmetric atrial morphogenesis [1, 28]. Mouse knockout models have shown it to suppress pathways for sinoatrial node formation [29, 30].

SNP rs2106261 on chromosome 16q22 has an odds ratio of 1.44 for atrial fibrillation (P = 1.6 X 10^{-11}) in persons of European descent [20], and occurs in approximately 20% of that population [19]. This SNP is located around zinc finger homeobox 3 (ZFHX3) gene [19], which encodes a transcription factor that acts as a regulator of alpha-fetoprotein expression [19, 31]. Its role in cardiac or pulmonary tissue physiology remains unclear.

A recent GWAS focusing on lone atrial fibrillation found that the SNP rs13376333 on chromosome 1q21 was associated with lone atrial fibrillation in persons of European ancestry. Carriers of the minor allele of this SNP were at 50% increased risk for developing lone atrial fibrillation: OR, 1.52 (95% CI, 1.40 - 1.64; P = 1.83 X 10^{-21}) [19, 22]. This SNP is located between the first and second exons of *KCNN3* [19], a calcium-activated, small conductance potassium channel, and its involvement in rabbit models of pulmonary venous ectopy provide a plausible biological pathway for the development of atrial fibrillation [32]. Furthermore, six additional variants have recently been discovered by Ellinor et al. and although the reported odds ratios were modest, these associations achieved genome-wide significance [47].

3. Hypothesis

Our primary hypothesis is that a risk score comprised of approximately 10 SNPs that are associated with atrial fibrillation at the GWAS level is associated with the development of atrial fibrillation among previously undiagnosed patients at high risk for atrial fibrillation. A current example of these SNPs is shown in Table 1. As a secondary hypothesis, we will test the association between atrial fibrillation diagnosed in this study with a subset of SNPs reported to be associated with atrial fibrillation and with fine-mapping SNPs. We will also test the association between atrial fibrillation of less than and greater than 30 seconds and a panel of approximately 10 SNPs.

We propose to perform targeted genotyping of patients at high risk for atrial fibrillation who are referred for ambulatory cardiac rhythm monitoring. To potentially increase our diagnostic yield, cardiac rhythm monitoring may be performed for up to two weeks using a prolonged-duration ambulatory ECG recording device such as the iRhythm Zio® Patch or the Sorin Spider Flash-t™ loop recorder.

Table 1.

rs#	Locus	Gene	OR source	OR (95% CI)	AF
rs2200733	4q25	PITX2	[1] and [25]	1.76 (1.49-2.07)	0.115
rs10033464	4q25	PITX2	[1] and [25]	1.32 (1.23-1.42)	0.097
rs2106261	16q22	ZFHX3	[47]	1.24 (1.17-1.30)	0.181
rs13376333	1q21	KCNN3	[47]	1.18 (1.13-1.23)	0.336
rs3903239	1q24	PRRX1	[47]	1.14 (1.10-1.17)	0.448
rs3807989	7q31	CAV1	[47]	0.90 (0.87-0.92)	0.42
rs10821415	9q22	C9orf3	[47]	1.11 (1.08-1.15)	0.397
rs10824026	10q22	SYNPO2L	[47]	0.87 (0.83-0.91)	0.165
rs1152591	14q23	SYNE2	[47]	1.13 (1.09-1.17)	0.487
rs7164883	15q24	HCN4	[47]	1.19 (1.14-1.24)	0.168

4. Study Design

This will be a multi-center, bi-national prospective study involving patients who are at high risk for atrial fibrillation and are referred for ambulatory cardiac rhythm monitoring or have a first diagnosis of atrial fibrillation by electrocardiogram (ECG) on the day of their enrollment. .

Patients defined, as high risk for atrial fibrillation will be persons with:

1. No prior history of atrial fibrillation and/or atrial flutter (AFIB/AF) - with symptoms of high clinical suspicion for AFIB/AF prompting referral for ambulatory cardiac rhythm monitoring to evaluate for potential AFIB/AF, and
2. Any of the following features: ischemic stroke or TIA with no defined etiology (In prior 6 months) [3, 4, 6, 7], hypertension [33], increased body mass index (BMI >30kg/m^2) [33], heart failure [33], clinically significant murmur [33], prolonged PR interval on resting ECG (>200msec) [33], chronic kidney disease [34], hypertrophic cardiomyopathy [35], congenital heart disease [36], chronic obstructive pulmonary disease [37, 38], sleep apnea [39-41], thyroid disease [42, 43], family history of atrial fibrillation [44], diabetes [45] or excess alcohol consumption (Male > 14 drinks/week, Female >7 drinks/week)[46].

Patients will be 40 years or older and capable of providing informed consent, providing a blood sample and wearing a Zio® Patch for up to 14 days or a Sorin SpiderFlash-t™ for up to 21 days. Each patient will be identified by a unique study number (e.g. 001) and their initials. (Patient initials will <u>not</u> be listed on the sample tubes.) Patients who receive the Zio® Patch or the SpiderFlash-t™ will be registered on their respective websites by their study ID.

Atrial fibrillation or flutter is defined using standard electrographic criteria and a minimum event duration of 5 seconds [3, 6].

Physicians and/or medical centers that routinely investigate patients at high risk for atrial fibrillation will be selected for participation. The care team will identify eligible patients for this study at the time of their medical assessment. Additionally, patients may also be identified when they present as 'walk ins' to the cardiac investigations laboratory. All potential patients will be provided with an informed consent form. If a patient is then agreeable to inclusion in the study a coordinator will then take the patient to a private room, inform them of the study details, and provide them with adequate time to read the informed consent form (ICF). The study coordinator

will review the ICF with the patient and answer all of their questions. The patient will sign the ICF and the coordinator and the patient will proceed to the cardiac rhythm monitoring lab and/or the laboratory. Patients who presented with symptoms and a diagnosis of atrial fibrillation was confirmed at the time of the visit by an (ECG) will not be fitted with a monitoring device but provide blood for genetic testing and future research. Patients who otherwise meet inclusion/exclusion criteria will be fitted by the technician with a Zio® Patch or SpiderFlash-t™ loop recorder. All patients will provide a blood sample (in a 4 ml EDTA tube) for genotyping and a serum sample (in 2 x 10ml serum separating tubes) for future research. (These patients may also be fitted with a traditional Holter monitor).

iRhythm Technologies Zio® Patch

If the patient is fitted with a Zio® Patch they will be provided the Patient Instructions and Diary booklet (Appendix 1) with instructions for recording any symptoms experienced while wearing the patch. They will also receive the Zio® Patch Return Box - a pre-paid box for return of the Zio® Patch to iRhythm Technologies. The patient will continue to wear the Zio® Patch for up to 14 days. Upon completion, the patient will remove the device and return it to the device manufacturer by prepaid box, for data processing and generation of a report of the findings.

The results of Zio® Patch monitoring will be analyzed by the staff of iRhythm Technologies, Inc. The data from the Zio® Patch will not be relied upon to make timely clinical diagnoses, but rather the information obtained will be used to help us more accurately assess our approach to treating these patients.

Upon review of the report, an iRhythm representative will notify the principal investigator by phone and follow up e-mail if there are any significant findings. (See Appendix 2 for Zio® Patch Notification Criteria.)

The site investigators will be notified by e-mail when there is a new report available on the website for his review. (See Appendix 3 for Zio® Patch Sample Report.) The site investigators will log-on with a unique ID, review the report, add comments, and sign off on the report.

Sorin SpiderFlash-t™ Loop Recorder

Patients enrolled in British Columbia will be outfitted with a Sorin SpiderFlash-t™ Loop Recorder, an automatic and patient-activated long-term cardiac loop recorder. In addition to being patient activated, the Spider Flash is automatically programmed to activate recording if it senses atrial fibrillation. The recorder is water- and shock-resistant, and is supplied with a disposable water resistant plastic holster that hangs around the patient's neck. A patient diary with ECG connection instructions is provided to assist the patient with replacement of the recording electrodes to allow for bathing and skin care during the time the patient is wearing the recorder.

Events that are considered possible occurrences of atrial fibrillation will be adjudicated by a physician with expertise in cardiac rhythm interpretation. Primary end point events will be instances of atrial fibrillation/flutter.

5. Sample Storage and Analysis

A blood sample (in a 4 ml EDTA tube) for genotyping and a serum sample (in two 10ml red top tubes) for future research will be obtained from each patient.

Blood samples will be delivered to the site's laboratory or storage area. The serum sample will be centrifuged and the serum stored in a -80° freezer. (If no -80°freezer is available, sites may store the samples in a -20° freezer, as long as it is not a "frost-free" freezer.) The blood sample will be stored in a -80° or a -20° "frost free" freezer. The blood and serum samples will be stored sent via overnight courier to Celera, Alameda, approximately one time per month.

Celera will perform Genotyping on subject blood samples and DNA will be stored at Celera for future research. Serum samples will be stored at Quest Diagnostics for use in future research.

Sites will receive a "Laboratory Reference Manual" for instructions on sample collection and storage.

Future research

By signing the informed consent and enrolling in the study, patients are consenting to the genotyping of their blood sample for the purposes of exploring the association between the identified SNPs and the risk of developing atrial fibrillation.

Patients will be given the option to opt out of future research by placing a check mark before the following statement in the informed consent:

> ___ Check here if you **do not** want your sample used for **future cardiovascular disease research.**

If patients agree to future research, samples and subject information will be kept for up to 20 years. In 2033, the code that links the sample to the subject's traditional identifying information will be destroyed.

6. Statistical Considerations

SNP effect sizes and frequencies were determined from the literature and the International HapMap Project database as indicated in Table 1. Expected occurrences of atrial fibrillation in the two groups were calculated using reference to previous studies as outlined above [7, 8, 33]. We expect 80 atrial fibrillation events in a 1000 high-risk patients [48, 49]. Using these event rates and an alpha error of 5%, the power to detect an association between a 10-SNPs risk score and atrial fibrillation is >90%. The power to detect association between a 4-SNP risk score and atrial fibrillation is >80%.

7. Inclusion and Exclusion Criteria

Inclusion Criteria:

- **(1) Symptoms of high clinical suspicion for atrial fibrillation prompting referral for ambulatory cardiac rhythm monitoring for potential atrial fibrillation OR (2) initial presentation and diagnosis of atrial fibrillation with in-office or in patient ECG on the day of enrollment.**

  AND

- At high risk for atrial fibrillation, defined as any one of the following: ischemic stroke or TIA with no defined etiology (in prior 6 months) [3, 4, 6, 7], hypertension [33], increased body mass index (BMI >30kg/m^2) [33], heart failure [33], clinically significant murmur [33], prolonged PR interval on resting ECG [33], chronic kidney disease [34], hypertrophic cardiomyopathy [35], congenital heart disease [36], chronic obstructive pulmonary disease [37, 38], sleep apnea [39-41], thyroid disease [42, 43], family history of atrial fibrillation [44], diabetes [45] or excess alcohol consumption (Male > 14 drinks/week, Female >7 drinks/week)[46].
- Age 40 years or older
- Capable of providing informed consent
- Capable of wearing a Zio Patch for up to 14 days or Sorin SpiderFlash-t™ loop recorder for up to 21 days.
- Capable of providing a blood sample

Exclusion Criteria:

- Previously documented atrial fibrillation or atrial flutter.
- Prior cardiac surgery (coronary artery bypass grafting, valve replacement or repair, pericardial stripping, etc) within the past 30 days.
- Hyperthyroidism.
- Have known skin allergies, conditions, or sensitivities (e.g. allergy to adhesives, psoriasis) as the Zio Patch should not be used on patients with known skin allergies, conditions, or sensitivities.
- Are receiving pacing therapy.
- Are anticipated to receive or require external cardiac defibrillation during the monitoring period.
- Are anticipated to have exposure to high frequency surgical equipment during the monitoring period.

8. Possible Benefits

- Providing physicians data regarding the occurrence of arrhythmias for which patients may be asymptomatic and thus not observed by traditional 24-hour Holter monitoring, the knowledge of which may be of help in formulating treatment plans.
- Providing a potentially greater understanding of the characteristics (e.g. frequency, duration, rate, time of day) of arrhythmia episodes that are or are not associated with symptoms.

9. Possible Risks and Analysis of Risk/Benefit Ratio

In comparison with traditional outpatient cardiac rhythm monitoring, no new risks will be introduced by the study. It is anticipated that any complications with the Zio® Patch or Sorin SpiderFlash-t™ would be comparable to those already identified with commercially available devices used for traditional outpatient cardiac rhythm monitoring.

There is minimal risk to a patient receiving cardiac rhythm monitoring and, in general, complications for the Zio® Patch are related to the adhesive that affixes the patch to the subject. The Zio® Patch has been cleared for use by the FDA for patients who do not have known skin allergies or a family history of skin allergies. Company experience for skin allergies in 2011 is 0.02% in over 30,000 patients. Additional risks to the patient include the potential for emotional or mental discomfort of knowingly wearing a cardiac rhythm monitor for an extended period of time. We consider this risk to be quite low.

There is also minimal risk to a patient using the loop recorder and is confined to slight skin irritation where the electrodes are adhered to the skin.

In spite of all the safety measures that we will use, we cannot guarantee that the subject's identity will never become known. Although genetic information is unique to each subject, individuals do share some genetic information with their children, parents, siblings or other blood relatives. Consequently, it may be possible that genetic information from them could be used to help identify the study subject. Similarly, it may be possible that genetic information from the study subject could be used to help identify them. No personal data regarding analysis of the blood samples will be given back to the study subject.

10. Risk Management Procedures

Data Safety and Monitoring Board (DSMB)

A DSMB will be assembled to provide an independent safety review of adverse events (AEs) and serious adverse events (SAEs) associated with the study. The DSMB will be comprised of individuals with expertise in cardiovascular disease, multi-center clinical trials and biostatistics:

> Steven Steinhubl, *M.D., M.S.*: clinical trialist who has spearheaded multiple multi center clinical trials, currently the Director of Digital Medicine at the Scripps Translational Science Institute, previously at Geisinger Health in Pennsylvania.
>
> Cinnamon Bloss, *Ph.D.*: clinical psychologist, Director of Social Sciences and Bioethics at the Scripps Translational Research Institute and clinical trialist.
>
> Jill Waalen, *M.D., M.P.H., F.A.C.P.M*.: medical epidemiologist, biostatistician and Diplomat of the American Board of Preventative Medicine.

It is anticipated that the AEs associated with the study may include infection or irritation at the site of the phlebotomy, other complications from the blood draw, allergic reaction to the Zio patch adhesive and potential for loss of privacy and/or confidentiality.

The DSMB will review the safety data including all reported adverse events and SAE's after every 200 patients. At any time should safety concerns arise, the DSMB may convene an ad hoc meeting or make recommendations to the Sponsor about the appropriateness of continuing the study.

In addition to their routine safety review, the DSMB will assess the study's cumulative enrollment, and primary and secondary event rates in order to make appropriate recommendations on expected trial length, enrollment abnormalities or site-specific deviation from the protocol.

Loss of Privacy

Only the investigator and research staff at the patient's site will have access to the patient's fully identified medical information. The information that matches the code to the identifying information will be kept in a safeguarded database that is password protected and any paper files will be kept in a secure location at the enrolling research site.

All blood samples will be labeled with a unique bar-code study subject number. This code will be used to identify these biological samples throughout their processing and storage in the research laboratory. There will be no traditional identifying information, such as name, birth

date, or social security number included with the biological samples. Only the site will have the information that matches the code to traditionally used identifying information.

Information from analysis of the coded samples and de-identified medical information will be placed in a controlled-access database on the internet. The database used for analysis will contain genotypes and clinical variables (listed in Appendices 4 and 5) and be coded so as not contain any traditionally used identifying information that could be used to identify the patient, and Dr. Topol will share these coded data sets with study non-Scripps co-investigators and their research staff.

A new Federal law, called the Genetic Information Nondiscrimination Act (GINA), generally makes it illegal for health insurance companies, group health plans, and most employers to discriminate against subjects based on their genetic information. This law generally will protect subjects in the following ways:

- Health insurance companies and group health plans may not request their genetic information that we get from this research.
- Health insurance companies and group health plans may not use their genetic information when making decisions regarding their eligibility or premiums.
- This new Federal law does not protect subjects against genetic discrimination by companies that sell life insurance, disability insurance, or long-term care insurance.

11. Recruitment

Patients at high risk for atrial fibrillation will be identified during or prior to their appointment at the cardiac investigations laboratory.

12. Subject Payment/Costs

Subjects will not be remunerated for participation in the study. There is no cost to the subject for study participation.

13. Estimated Duration of Study

The study will be conducted at multiple participating research sites in order to meet the study enrollment goals in a timely manner. If the endpoint of 80 atrial fibrillation/flutter events have not been met at the end of 2 years, the timeline and enrollment may be extended until 80

endpoint (atrial fibrillation/flutter) events are recorded. If 80 endpoint events have occurred before 650 patients are enrolled, enrollment may be terminated before 650 patients are enrolled.

14. Consent Procedures

Study purpose, methods, materials, risks, benefits, and alternatives will be discussed by the study coordinator at the time of their scheduled outpatient visit. Informed consent will be obtained in writing and documented by the study coordinator's and patient's signature on the attached form.

15. Privacy

All information regarding the study will be discussed in private consultation. Distribution and instructions for use and return of the Zio® Patch will be provided at the time of the outpatient visit. The results of the monitoring will be discussed by telephone or at a scheduled outpatient visit, consistent with current practice.

Subjects will not be made aware of their genotype status.

16. Data Security

The collection and processing of personal data from subjects enrolled in this study will be limited to the data needed to investigate this study's hypothesis and the clinical data typically used as covariates in the analysis of cardiovascular clinical studies. Access to data will be limited to those authorized by Principal Investigator and study sponsor.

Scripps Translational Science Institute affirms the subject's right to protection against invasion of privacy. Data files are stored on a password-protected computer/database and will be accessible only to the above listed investigators and research staff. Subjects blood samples and data will be assigned and identified by a unique code. Traditional identifying information such as name, address, phone, medical record number, etc. will not be included in the database. Only the site investigators and the research staff will have the link that can match the code to traditional identifying information. Study files will be kept in secure locations at each study site. The data sets used for analysis will be coded and not contain any traditionally used identifying information that could be used to identify the patient.

17. Subject Withdrawal

Subject participation is voluntary and the subject can discontinue participation at any time without loss of benefits or penalty. A subject who wishes to withdraw consent should make a request in writing to the Principal Investigator. Any data that has been entered in the database and samples that have already been processed will be included in the analysis of the study. Any remaining data and samples will not be used in future research. No data will be collected until after full consent has been obtained. Subjects who withdraw consent or are found ineligible will be replaced until the study endpoint is met.

18. Monitoring

In accordance with the FDA Guidance for Industry, Oversight of Clinical Investigations – A Risk-Based Approach to Monitoring (August 2013), STSI will monitor the study throughout its duration by way of remote monitoring,

Remote monitoring of data will be accomplished through comparison of:

- Dates of study procedures with date of informed consents on eCRFs.
- Patient ID (barcode), initials, age, date of blood sample, and gender on eCRF with Test Request Form.
- Patient ID (barcode), initials and date of blood sample with eCRF. And SampleCollection Log.
- Number of tubes sent (on Sample Shipment Form) with Sample Collection Log and eCRF.
- Patient ID (barcode), initials, gender, age and date of Zio® Patch placement with Zio report and eCRF.
- In order to verify source documents, for the first 5 patients enrolled, site staff will be asked to copy the 4-page case report forms (CRFs) and signature page of the patient's Informed Consent form and send to Scripps when the laboratory documents are sent (approximately monthly). Periodically throughout the study, Scripps will also ask sites to verify eligibility of approximately 20% of enrolled subjects by sending or faxing de-identified medical records which document that enrolled patients had symptoms of high clinical suspicion for atrial fibrillation prompting referral for ambulatory cardiac rhythm monitoring for potential atrial fibrillation and were at high risk for atrial fibrillation.

All blood collection-related adverse events and/or privacy issues will be reported to the sponsor. STSI will collect the appropriate regulatory documentation from each clinical site according to ICH/GCP guidelines.

Enrollment may not begin at the study site until all required documentation is collected, key study personnel are trained and written approval from STSI is given to start enrollment.

19. Record Retention

Research records will be kept for at least 20 years after study completion. Scripps will continue to store and use the subject information for up to 20 years. In 2033, the code that links the subject to the identifying health and trait information will be destroyed. The sample and related de-identified health and trait information may be kept indefinitely. Record retention must also comply with any specific requirements of the local reviewing IRB. (i.e., Scripps- must keep HIPAA form for at least 6 years after study completion)

Records will ultimately be kept at the research site until STSI has given written permission to destroy the records. If record retention cannot be maintained by the participating sites, STSI must be notified and storage will be provided.

20. Publication

The results of this research may be presented at meetings or in publication. However, the subject's identity will not be disclosed in those presentations.

21. Facilities and Personnel

Site Selection

Investigators and participating centers will be selected at institutions with access to an established IRB or equivalent that meets on a regular basis. An interest in participating in the study and willingness to support monitoring and auditing procedures will be prerequisites. Other considerations include the center's ability to execute the study protocol and a prospective patient population that will meet the protocol criteria. The Principal Investigator and the other physician investigators will be qualified practitioners and will be experienced in the diagnosis of subjects with cardiovascular conditions.

Clinical Trial Agreement

A Clinical Trial Agreement must be signed by all parties named in the Clinical Trial Agreement and returned to Scripps Translational Science Institute (Department of Genomic Medicine) prior to the commencement of any study activities.

Personnel Training

Center personnel (investigators, coordinators, and other key clinical staff) will be trained by the sponsor on the study protocol and study activities relevant to their role prior to conducting any study activities. Training will also include but is not limited to blood collection and transport, case report form (CRF) completion, and use of an electronic data capture program.

It is the responsibility of the study site investigator to ensure that there is adequate staff to complete the study according to the protocol and the entire research staff designated to this study has the education and the training required to perform the proposed procedures, and the procedures are allowed under the scope of practice.

Center Readiness

All local regulatory requirements need to be fulfilled and each study center must have written documentation of center/investigator readiness provided by Scripps Translational Science prior to enrolling any subjects in the study.

Bibliography

1. Gudbjartsson, D.F., et al., Variants conferring risk of atrial fibrillation on chromosome 4q25. Nature, 2007. 448(7151): p. 353-7.
2. Go, A.S., et al., Prevalence of diagnosed atrial fibrillation in adults: national implications for rhythm management and stroke prevention: the AnTicoagulation and Risk Factors in Atrial Fibrillation (ATRIA) Study. JAMA, 2001. 285(18): p. 2370-5.
3. Healey, J.S., et al., Subclinical atrial fibrillation and the risk of stroke. N Engl J Med, 2012. 366(2): p. 120-9.
4. Wolf, P.A., et al., Epidemiologic assessment of chronic atrial fibrillation and risk of stroke: the Framingham study. Neurology, 1978. 28(10): p. 973-7.
5. Wolf, P.A., R.D. Abbott, and W.B. Kannel, Atrial fibrillation: a major contributor to stroke in the elderly. The Framingham Study. Arch Intern Med, 1987. 147(9): p. 1561-4.
6. Tayal, A.H., et al., Atrial fibrillation detected by mobile cardiac outpatient telemetry in cryptogenic TIA or stroke. Neurology, 2008. 71(21): p. 1696-701.
7. Jabaudon, D., et al., Usefulness of ambulatory 7-day ECG monitoring for the detection of atrial fibrillation and flutter after acute stroke and transient ischemic attack. Stroke, 2004. 35(7): p. 1647-51.
8. Liao, J., et al., Noninvasive cardiac monitoring for detecting paroxysmal atrial fibrillation or flutter after acute ischemic stroke: a systematic review. Stroke, 2007. 38(11): p. 2935-40.
9. Sacco, R.L., et al., Infarcts of undetermined cause: the NINCDS Stroke Data Bank. Ann Neurol, 1989. 25(4): p. 382-90.
10. Petty, G.W., et al., Ischemic stroke subtypes: a population-based study of incidence and risk factors. Stroke, 1999. 30(12): p. 2513-6.
11. Kolominsky-Rabas, P.L., et al., Epidemiology of ischemic stroke subtypes according to TOAST criteria: incidence, recurrence, and long-term survival in ischemic stroke subtypes: a population-based study. Stroke, 2001. 32(12): p. 2735-40.
12. Schulz, U.G. and P.M. Rothwell, Differences in vascular risk factors between etiological subtypes of ischemic stroke: importance of population-based studies. Stroke, 2003. 34(8): p. 2050-9.
13. Schneider, A.T., et al., Ischemic stroke subtypes: a population-based study of incidence rates among blacks and whites. Stroke, 2004. 35(7): p. 1552-6.
14. Lee, B.I., et al., Yonsei Stroke Registry. Analysis of 1,000 patients with acute cerebral infarctions. Cerebrovasc Dis, 2001. 12(3): p. 145-51.

15. Glotzer, T.V., et al., Atrial high rate episodes detected by pacemaker diagnostics predict death and stroke: report of the Atrial Diagnostics Ancillary Study of the MOde Selection Trial (MOST). Circulation, 2003. 107(12): p. 1614-9.
16. Glotzer, T.V., et al., The relationship between daily atrial tachyarrhythmia burden from implantable device diagnostics and stroke risk: the TRENDS study. Circ Arrhythm Electrophysiol, 2009. 2(5): p. 474-80.
17. Olesen, J.B., et al., Risks of thromboembolism and bleeding with thromboprophylaxis in patients with atrial fibrillation: A net clinical benefit analysis using a 'real world' nationwide cohort study. Thromb Haemost, 2011. 106(4): p. 739-49.
18. Lubitz, S.A., et al., Association between familial atrial fibrillation and risk of new-onset atrial fibrillation. JAMA, 2010. 304(20): p. 2263-9.
19. Lubitz, S.A., et al., Genetics of atrial fibrillation: implications for future research directions and personalized medicine. Circ Arrhythm Electrophysiol, 2010. 3(3): p. 291-9.
20. Benjamin, E.J., et al., Variants in ZFHX3 are associated with atrial fibrillation in individuals of European ancestry. Nat Genet, 2009. 41(8): p. 879-81.
21. Gudbjartsson, D.F., et al., A sequence variant in ZFHX3 on 16q22 associates with atrial fibrillation and ischemic stroke. Nat Genet, 2009. 41(8): p. 876-8.
22. Ellinor, P.T., et al., Common variants in KCNN3 are associated with lone atrial fibrillation. Nat Genet, 2010. 42(3): p. 240-4.
23. Shi, L., et al., Assessment of association of rs2200733 on chromosome 4q25 with atrial fibrillation and ischemic stroke in a Chinese Han population. Hum Genet, 2009. 126(6): p. 843-9.
24. Husser, D., et al., Chromosome 4q25 variants and atrial fibrillation recurrence after catheter ablation. J Am Coll Cardiol, 2010. 55(8): p. 747-53.
25. Kaab, S., et al., Large scale replication and meta-analysis of variants on chromosome 4q25 associated with atrial fibrillation. Eur Heart J, 2009. 30(7): p. 813-9.
26. Viviani Anselmi, C., et al., Association of rs2200733 at 4q25 with atrial flutter/fibrillation diseases in an Italian population. Heart, 2008. 94(11): p. 1394-6.
27. Body, S.C., et al., Variation in the 4q25 chromosomal locus predicts atrial fibrillation after coronary artery bypass graft surgery. Circ Cardiovasc Genet, 2009. 2(5): p. 499-506.
28. Franco, D. and M. Campione, The role of Pitx2 during cardiac development. Linking left-right signaling and congenital heart diseases. Trends Cardiovasc Med, 2003. 13(4): p. 157-63.
29. Faucourt, M., et al., The pitx2 homeobox protein is required early for endoderm formation and nodal signaling. Dev Biol, 2001. 229(2): p. 287-306.

30. Mommersteeg, M.T., et al., Molecular pathway for the localized formation of the sinoatrial node. Circ Res, 2007. 100(3): p. 354-62.
31. Morinaga, T., et al., A human alpha-fetoprotein enhancer-binding protein, ATBF1, contains four homeodomains and seventeen zinc fingers. Mol Cell Biol, 1991. 11(12): p. 6041-9.
32. Ozgen, N., et al., Early electrical remodeling in rabbit pulmonary vein results from trafficking of intracellular SK2 channels to membrane sites. Cardiovasc Res, 2007. 75(4): p. 758-69.
33. Schnabel, R.B., et al., Development of a risk score for atrial fibrillation (Framingham Heart Study): a community-based cohort study. Lancet, 2009. 373(9665): p. 739-45.
34. Watanabe, H., et al., Close bidirectional relationship between chronic kidney disease and atrial fibrillation: the Niigata preventive medicine study. Am Heart J, 2009. 158(4): p. 629-36.
35. Glancy, D.L., et al., Atrial fibrillation in patients with idiopathic hypertrophic subaortic stenosis. Br Heart J, 1970. 32(5): p. 652-9.
36. Tikoff, G., A.M. Schmidt, and H.H. Hecht, Atrial fibrillation in atrial septal defect. Arch Intern Med, 1968. 121(5): p. 402-5.
37. Davidson, E., et al., Atrial fibrillation. Cause and time of onset. Arch Intern Med, 1989. 149(2): p. 457-9.
38. Buch, P., et al., Reduced lung function and risk of atrial fibrillation in the Copenhagen City Heart Study. Eur Respir J, 2003. 21(6): p. 1012-6.
39. Gami, A.S., et al., Association of atrial fibrillation and obstructive sleep apnea. Circulation, 2004. 110(4): p. 364-7.
40. Kanagala, R., et al., Obstructive sleep apnea and the recurrence of atrial fibrillation. Circulation, 2003. 107(20): p. 2589-94.
41. Schulz, R., H.J. Eisele, and W. Seeger, Nocturnal atrial fibrillation in a patient with obstructive sleep apnoea. Thorax, 2005. 60(2): p. 174.
42. Woeber, K.A., Thyrotoxicosis and the heart. N Engl J Med, 1992. 327(2): p. 94-8.
43. Sawin, C.T., et al., Low serum thyrotropin concentrations as a risk factor for atrial fibrillation in older persons. N Engl J Med, 1994. 331(19): p. 1249-52.
44. Palatini, P., Parental atrial fibrillation as a risk factor for atrial fibrillation in offspring. JAMA, 2004. 292(10): p. 1174-5.
45. Dublin, S., et al., Diabetes mellitus, glycemic control, and risk of atrial fibrillation. J Gen Intern Med, 2010. 25(8): p. 853-8.
46. Ettinger, P.O., et al., Arrhythmias and the "Holiday Heart": alcohol-associated cardiac rhythm disorders. Am Heart J, 1978. 95(5): p. 555-62.

47. Ellinor P et al., Meta-analysis identifies six new susceptibility loci for atrial fibrillation. Nat Genet. 2012 29;44(6):670-5

48. Bhatt A et al., Predictors of occult paroxsysmal atrial fibrillation in cryptogenic strokes detected by long-term noninvasive cardiac monitoring. Stroke Res Treat. 2011 22;2011p172074.

49. Schnabel RB et al., Development of a risk score for atrial fibrillation (Framingham Heart Study): a community based cohort study. Lancet 2009 28;373(9665):7339-45.
