# Supplementary material for: Validation of a genetic risk score for atrial fibrillation: A prospective multicenter cohort study
Source: PLoS Med. 2018 Mar 13;15(3):e1002525. doi: 10.1371/journal.pmed.1002525 (PMC5849279; doi:10.1371/journal.pmed.1002525)
Supplement: S1 Table — (PDF) [file pmed.1002525.s004.pdf]

S1 Table: US and Canadian patient recruitment centers

Scripps Green Hospital / Scripps Clinic: La Jolla, California (Dr. David Rubenson and Dr. Doug Gibson)

Clearwater Cardiovascular and Interventional Consultants: Clearwater, Largo and Safety Harbor, Florida (Dr. John Garner)

Advanced Cardiovascular, LLC: Alexander City and Auburn, Alabama. (Dr. Brian Foley)

PharmaTex Research: Amarillo, Texas. (Dr. David Brabham)

University of California – San Francisco Medical Center: San Francisco, California (Dr. Gregory Marcus)

Benaroya Research Institute at Virginia Mason: Seattle, Washington. (Dr. Robert Rho)

North Shore University Health System Medical Group: Bannockburn, Illinois. (Dr. Eli Lavie)

Vanderbilt University Medical Center: Nashville, Tennessee. (Dr. George Crossley)

Nebraska Heart Institute: Hastings, Nebraska. (Dr. Peter Gallagher)

Nebraska Heart Institute: Lincoln, Nebraska. (Dr. Erich Fruehling)

KentuckyOne Health Medical Group - Jewish Hospital: Louisville, Kentucky (Dr. Naresh Solankhi)

KentuckyOne Health Medical Group - St. Joseph Health System: Lexington, Kentucky. (Dr. Michael Schaeffer)

Memorial Heart Institute: Chattanooga, Tennessee (Dr. David Wendt)

Tallahasssee Research Institute: Tallahassee, Florida. (Dr. Gad Silberman)

Royal Jubilee Hospital: Victoria, British Columbia, Canada. (Dr. Andrew Penn)
