# Supplementary material for: Validation of a genetic risk score for atrial fibrillation: A prospective multicenter cohort study
Source: PLoS Med. 2018 Mar 13;15(3):e1002525. doi: 10.1371/journal.pmed.1002525 (PMC5849279; doi:10.1371/journal.pmed.1002525)
Supplement: S2 Table — (PDF) [file pmed.1002525.s005.pdf]

S2 Table: Performance of the Individual SNPs of the AF-GRS.

Locus	Gene	SNP	MAF	Risk Estimate	OR (95% CI)	p-value
1q21	KCNN3	rs13376333	0.298	1.13	1.18 (0.84,1.65)	0.35
1q24	PRRX1	rs3903239	0.404	1.14	1.35 (0.98,1.86)	0.06
4q25[1]	PITX2	rs10033464	0.101	1.39	0.27 (0.11,0.67)	0.005
4q25[1]	PITX2	rs17570669	0.065	0.73	1.42 (0.82,2.46)	0.21
4q25[2]	PITX2	rs2200733	0.128	1.72	1.61 (1.09,2.39)	0.02
4q25[1]	PITX2	rs3853445	0.272	0.86	0.93 (0.64,1.34)	0.70
7q31	CAV1	rs3807989	0.421	0.90	0.93 (0.68,1.29)	0.67
9q22	C9orf3	rs10821415	0.398	1.11	1.46 (1.07,1.98)	0.02
10q22	SYNPO2L	rs10824026	0.180	0.87	0.67 (0.43,1.06)	0.08
14q23	SYNE2	rs1152591	0.459	1.13	1.43 (1.04,1.95)	0.03
15q24	HCN4	rs7164883	0.189	1.19	1.64 (1.13,2.37)	0.009
16q22	ZFHX3	rs2106261	0.189	1.24	1.35 (0.92,2.00)	0.13

Model not adjusted by clinical factors. MAF, mean allele frequency; OR, odds ratio; CI, confidence interval. Risk Estimates were obtained from the literature. p-values presented without correction for multiple testing.

[1]Adjusted for rs2200733

[2]Adjusted for rs10033464
