# Supplementary material for: Validation of a genetic risk score for atrial fibrillation: A prospective multicenter cohort study
Source: PLoS Med. 2018 Mar 13;15(3):e1002525. doi: 10.1371/journal.pmed.1002525 (PMC5849279; doi:10.1371/journal.pmed.1002525)
Supplement: S3 Table — (PDF) [file pmed.1002525.s006.pdf]

S3 Table: Risk estimates of AF events according to AF-GRS Quintile (9SNP model)

AF-GRS Quintile	Unadjusted OR (95% CI)	p-value	Adjusted* OR (95% CI)	p-value
**1**	Reference	--------	Reference	--------
**2**	1.49 (0.53-4.20)	0.45	1.60 (0.53-4.83)	0.40
**3**	**2.55** (1.02-6.36)	0.04	1.97 (0.76-5.17)	0.16
**4**	**4.20** (1.78-9.94)	0.001	**4.56** (1.84-11.33)	0.001
**5**	**4.72** (2.00-11.14)	<0.001	**5.07** (2.04-12.60)	<0.001

AF-GRS Quintile ranges: Q1 < 0.22; Q2 > 0.22 and < 0.36; Q3 > 0.36 and < 0.54; Q4 > 0.54 and < 0.79; Q5 > 0.79. * Adjusted by age, sex, smoking status, BMI, diabetes, hypertension, prior myocardial infarction and heart failure. AF-GRS, atrial fibrillation genetic risk score; OR, odds ratio; CI, confidence interval.
