# Supplementary material for: Validation of a genetic risk score for atrial fibrillation: A prospective multicenter cohort study
Source: PLoS Med. 2018 Mar 13;15(3):e1002525. doi: 10.1371/journal.pmed.1002525 (PMC5849279; doi:10.1371/journal.pmed.1002525)
Supplement: S4 Table — (PDF) [file pmed.1002525.s007.pdf]

S4 Table: Risk estimates for both 12-SNP and 9-SNP AF GRS, highest quintile compared to lowest quintile, limited to self-reported white (n = 837).

	Unadjusted	Adjusted*
12-SNP GRS	**2.81**<br>[1.20, 6.57]<br>p = 0.02	**3.10**<br>[1.27, 7.59]<br>p = 0.01
9-SNP GRS	**4.23**<br>[1.87, 9.58]<br>p = <0.001	**4.55**<br>[1.93, 10.72]<br>p = <0.001

Odds ratio, [95% CI], p-value.

*Adjusted by age, sex, smoking status, BMI, diabetes, hypertension, prior myocardial infarction
